# A Framework for Collaborative Curation of Neuroscientific Literature

**DOI:** 10.3389/fninf.2017.00027

**Published:** 2017-04-19

**Authors:** Christian O'Reilly, Elisabetta Iavarone, Sean L. Hill

**Affiliations:** Blue Brain Project, École Polytechnique Fédérale de LausanneGeneva, Switzerland

**Keywords:** literature curation, neural network modeling, ontology, thalamocortical loop, annotation tools

## Abstract

Large models of complex neuronal circuits require specifying numerous parameters, with values that often need to be extracted from the literature, a tedious and error-prone process. To help establishing shareable curated corpora of annotations, we have developed a literature curation framework comprising an annotation format, a Python API (NeuroAnnotation Toolbox; NAT), and a user-friendly graphical interface (NeuroCurator). This framework allows the systematic annotation of relevant statements and model parameters. The context of the annotated content is made explicit in a standard way by associating it with ontological terms (e.g., species, cell types, brain regions). The exact position of the annotated content within a document is specified by the starting character of the annotated text, or the number of the figure, the equation, or the table, depending on the context. Alternatively, the provenance of parameters can also be specified by bounding boxes. Parameter types are linked to curated experimental values so that they can be systematically integrated into models. We demonstrate the use of this approach by releasing a corpus describing different modeling parameters associated with thalamo-cortical circuitry. The proposed framework supports a rigorous management of large sets of parameters, solving common difficulties in their traceability. Further, it allows easier classification of literature information and more efficient and systematic integration of such information into models and analyses.

## Introduction

In the context of large-scale, highly detailed, and data-driven realistic modeling of the brain, developers are faced with the daunting task of reviewing voluminous, and ever growing, body of scientific papers to extract all information useful in constraining the large number of parameters involved in the modeling process. Without a rigorous approach to support this process, the extracted information is often not reusable outside of the project for which it has been built. Curated information from the literature that has been embedded into models are also often vulnerable to issues regarding the traceability of its origin. This happens for example when the embedding does not provide a means to trace back (1) the publication from which a numerical value has been extracted, (2) the exact place in the paper from where the information has been taken, or (3) the precise method used to transform published numbers into the values inserted into models. The last point is particularly important and applied transformations can take different forms. The most evident is unit conversion. But more subtle alterations are often applied such as changing the nature of the variable (e.g., passing from area to volume by considering hypotheses or supplementary factors, as it is the case when using cell counts per area from stereology studies to model neuronal volumetric densities) or combining different measures (e.g., taking the median of values reported by different sources).

In this paper, we present a collaborative framework for systematic curation of literature and creation of annotation corpora that aims at solving these issues. Corpora created through this system can be queried programmatically so that curated literature information can be integrated into modeling workflows in a systematic, reproducible, and traceable way. More specifically, we report on the development of an annotation format for scientific literature curation and on the public release of open-access tools to assist in the creation and management of annotation corpora. The presentation of the broader workflow, including the systematic integration of annotated information into modeling pipelines will be discussed in the sequel.

The proposed annotation system has been designed to allow, among other things, systematic annotation of numerical values reported in scientific publications so that experimental outcome can be efficiently synthesized and integrated into models. We demonstrate the usefulness of this approach by presenting an example of an open-access repository of annotations that has been created for modeling the thalamo-cortical loop in the context of the Blue Brain Project (EPFL, 2017).

### Terminology

The process of curating or annotating documents or datasets is defined in various ways depending on the context. To avoid confusion, we first define these concepts, as they are used in the current project.

By *literature curation*, we refer to the process in which the curator (i.e., the person performing the curation) identifies documents relevant to a specific topic and annotates (i.e., produces *annotations*) relevant information within these documents. An *annotation* is defined as a structured set of data specifying the precise localization of a subpart of a document which is of strategic interest. It is generally be supplemented with additional information (e.g., ontological terms describing in a formal way some characteristics of the annotated content). It may also contain a free-form comment to make explicit the relevance of the annotation, although such comments can be omitted if the highlighted part of the document is self-explanatory (e.g., “region X is connected to region Y with Z% probability”). Note that this process is significantly different from “annotating” as the (generally automated) process of extracting syntactic (e.g., part-of-speech) or morphological/semantic (e.g., identifying named entities) information from a text.

### Requirements

We established a set of requirements that a methodological framework for the curation and model-integration of the literature information should consider. These are presented in subsequent sections.

#### Collaborative workflow

The approach should allow for collaborative curation of a body of literature, meaning that annotations on a particular document can be made by different curators in a concurrent fashion. It must therefore be possible to easily merge produced annotations and to trace the history of modifications.

#### Reusable

The result of the curation process should be easily reusable by other researchers. It must therefore not rely on implicit knowledge of the curator. The important information associated with the annotations must be explicitly specified.

#### Easily machine-readable

The output of the curation process must be easily machine-readable. Although any computer file is “machine-readable,” what makes it easily readable is the use of a consistent formatting (e.g., CSV files, text files containing a well-defined JSON data structure) with fields using a highly consistent terminology (i.e., controlled vocabulary). This terminology should ideally by linked with identifiers from externally recognized entities (e.g., terms from public lexica or ontologies) allowing cross-referencing, indexing, and searching annotations in relation with specific concepts (e.g., species, brain regions, cell types, experimental paradigms). In that sense, free-form text fields are not easily machine-readable and should constitute only a limited part of the annotations.

#### Localizable

Annotations must be precisely and reliably localizable in the document of origin. This requires the specification of unique identifiers for annotated documents as well as the unambiguous localization, within the document, of the position and the extent of the annotated content.

#### User-friendly

The process of annotating a document must be as light as possible. The curation process is expected to be performed mainly by domain-experts, which are performing this task as part of other overarching goals. Therefore, it must not be perceived as implying a supplementary workload when compared to a more informal review of the literature. Not meeting this criterion is likely to result in poor user adoption and consequently, limited use of the proposed framework.

#### Integration with the existing software ecosystem

The design of the system should rest on well-established tools such that its design is simpler, requires less maintenance, and is more sustainable. It should also integrate with existing tools that might be used to produce annotations (e.g., text-mining tools) or to consume annotations (e.g., external user interfaces such as web-based neuroscience portals).

#### Support for modeling parameters

In order for this curation process to be useful in modeling projects, the proposed tool must provide the features necessary to annotate systematically and unambiguously numerical values reported from experiments.

#### Respect of legal environment

Annotations should be sharable without involving copyright issues. For example, they cannot be embedded in documents that are copyrighted.

### Existing solutions

Many projects have been conducted in the past years to support the annotation process in various contexts. For example, the online annotation service https://hypothes.is, proposes to add a supplementary layer to the Internet so that web pages can be directly annotated and commented (Perkel, 2015). WebAnno (Yimam and Gurevych, 2013) and BRAT (Stenetorp et al., 2012) are other examples of relevant projects but are more targeted along providing web-based collaborative environments for typical natural language processing tasks, such as annotating part-of-speech and syntactic dependencies, identifying named entities, etc. Other research teams have worked on developing pipelines for text-mining and automatic generation of annotation from papers (e.g., WhiteText French et al., 2015, Sherlok Richardet et al., 2015) or on manually annotating in great details corpora of scientific papers (e.g., the CRAFT Bada et al., 2012 and the GENIA Kim et al., 2003 corpora) to serve as benchmark in evaluating automated annotators. However, although these initiatives provide interesting tools to support the annotation process, none constitute a complete solution meeting our objectives: to provide an annotation framework which allows the collaborative construction of corpora containing literature-curated facts that can be integrated directly in models. Thus, these projects should not be seen as competitors or alternatives to the framework we are proposing. They are more complementary tools, which we aim to interface with, rather than replace.

## Design

### Collaborative structure

At the heart of this project is the idea to provide a simple, flexible, and collaborative framework for producing and reproducibly consuming literature annotations. For this reason, each publication is associated with one plain-text file containing the related annotations (i.e., it is a standoff format Thompson and McKelvie, 1997), as opposed, for example to a database-centric design or an in-text annotation system. These plain-text files, which structure is discussed in Section Annotation Format, are stored, versioned, and shared through GIT, a free and open-source distributed version control system. Aside from allowing easy sharing of annotation corpora through existing GIT servers (e.g., GitHub), it allows concurrent work on annotations, resolution of merging conflicts, and bookkeeping of modifications. Interaction with the GIT system has been made as transparent as possible to the user (e.g., automatic commit when changes to annotations are saved, dialog box asking if the modifications should be pushed to the server when exiting the application) although the underlying GIT repository can always be accessed directly in case of need.

### Ontologies

#### Use of standard ontologies

Annotations are tagged with terms from neuroscience ontologies to describe their context and allow the programmatic retrieval of subsets of annotations relevant to specific modeling or analysis objectives. Further, these tags constitute a direct bridge for interacting with third-party applications using the same ontologies.

However, although promising initiatives such as the Open Biomedical Ontologies (OBO) Foundry (Smith et al., 2007) have been put in place to promote good design practices, standardization, and interoperability, the world of ontologies is still a messy one. Many propositions are available with different, sometimes overlapping, coverage of the concepts related to neuroscience. Two common problems are the overdefinition of a concept (i.e., the same concept being partly or completely defined by different ontological terms) and its underdefinition (i.e., no ontological term defining completely or specifically a given concept). The first problem arises most acutely when trying to model a large field such as neuroscience by combining different ontologies, whose coverage overlaps (Ghazvinian et al., 2011). The second problem is intrinsic in modeling of an expanding knowledge involving dynamic creation of new concepts.

The proposed curation framework integrates terms from the SciCrunch resource registry (formerly the resources branch of Neurolex Larson and Martone, 2013) and the full Neuroscience Information Framework Standard Ontology (NIFSTD), and provides a large coverage of the neuroscience field through the integration of many domain-specific ontologies (Imam et al., 2012; Ozyurt et al., 2016; Grethe et al. personal communication). It also provides supports for integrating ontological terms from the Neuroinformatics Platform (NIP) of the Human Brain Project since it contains many terms useful for modeling neural networks (e.g., a comprehensive classification of cortical neurons). Integrating the NIFSTD and NIP ontologies is complicated by the huge size of these ontologies. This problem has been addressed by storing locally every term previously used, and fetching online new terms whenever required (see Supplementary Materials Section Integration of NIFSTD and NIP Ontologies for an extended discussion of this issue). Although using both ontologies may contribute to overdefining some terms, this effect is limited since both ontology services reuse some common third-party ontologies (e.g., the Allen Brain Institute ontology). Interacting with both NIP and NIFSTD ontologies is made easy by the fact that they share a very similar REST API (see O'Reilly, 2016a for an IPython Notebook example of programmatic interaction with these ontologies).

#### Definition of new terms

New ontological terms have been defined to complement existing ontologies only when no alternative was available. For example, the nomenclature of ionic currents was not fine-grained enough to be used to model neurons with a detailed electrophysiology like those of the Blue Brain Project (Markram et al., 2015). This use case required completing the existing hierarchy of ionic currents with new terms. In such cases, new terms have been defined in a separate CSV file (additionsToOntologies.csv) which is part of the *NeuroAnnotation Toolbox* (described below) source code. Providing such a mechanism for easily adding new terms is important for the flexibility of the system. However, these terms are not meant to constitute a separated ontology and will hopefully, at some point, be migrated toward more standard resources, such as the NIFSTD or NIP ontologies.

A more comprehensive effort has been undertaken in developing a controlled vocabulary for Modeling Parameter (MPCV) since no available resource was providing an adequate coverage for the framework proposed herein. Parameter types used here must be defined unambiguously and operationally with a sufficient level of granularity so that their annotated values can be directly used to instantiate model variables. Related ontologies such as the Computational Neuroscience Ontology (Le Franc et al., 2012) could be invaluable in adding a semantic level to the modeling parameters listed in the MPCV. However, they cannot be used directly in place of the MPCV because they do not currently provide the sufficient level of granularity. The descriptive level required by the proposed framework would be closer to the list of parameters defined in a project like NeuroElectro (Tripathy et al., 2014). However, this list is yet too limited^1^ to be directly reused for the use case described here. Further, from a practical point of view, the need of rapidly adding new terms to the MPCV would make the adoption of an external resource very cumbersome in the current phase of development. Thus, for reason of coverage, precision, and flexibility, MPCV terms have been specified directly in the NeuroAnnotation Toolbox source code as a separate CSV file (modelingDictionary.csv). Collectively, these terms are defined as a tree structure, which can be visualized online at https://github.com/BlueBrain/nat/blob/master/notebooks/parameterTree.png.

### Annotation format

**Figure 1:**
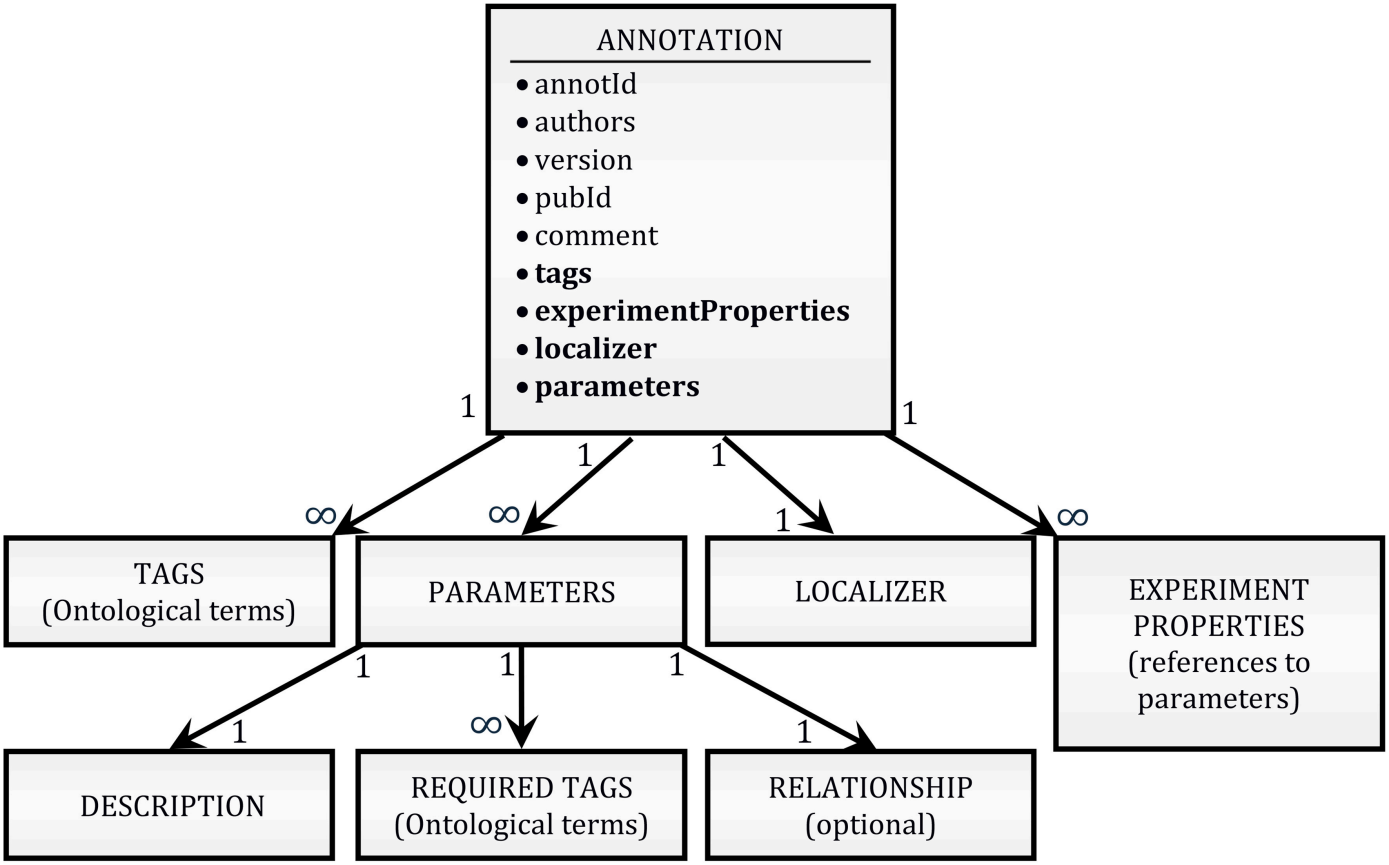
**High-level schema of the annotation format**. The block at the top of the schema (ANNOTATION) lists as bullet points all the fields of an annotation record. A thin font is used for simple attributes (e.g., strings); a bold font is used for attributes which are themselves complex JSON structures. The hierarchical relationships between the top-level object and its complex attributes are shown by arrows. The internal structure of the lower-level objects (PARAMETERS, LOCALIZER, etc.) has been omitted because both the hierarchical structure and the definition of the objects are context-dependent (e.g., there are 5 different types of LOCALIZER, with different internal structures; the variability of the hierarchical structure is due to compositionality of experimental values, as exemplified in Figure 2).

To provide an annotation format that is flexible enough to be adapted to future unforeseen needs of the community while remaining simple to read and write, we adopted a JSON serialization approach. Annotations for any given publication are written as a plain-text list of pretty-printed^2^ JSON strings (see **Figure 5D** for an example). A schema of the structure of an annotation is shown in Figure 1 (see O'Reilly, 2016b for a complete definition). In short, it contains mainly unique identifiers for the annotation and the publication, a list of tags, the identity of the authors of the annotation, the version of the annotation format, a free-form comment, a list of modeling parameters, a list of experiment properties, and a localizer. More explanation is given on the nature of some of these items below.

#### Unique identifiers

Every annotated publication is associated with a unique identifier. For that purpose, we use the DOI whenever one has been attributed to the publication. Otherwise, we set it to “PMID_” followed by the PubMed (NCBI, 2017) identification number (PMID) if the paper has been attributed one. Papers that are not referenced by PubMed and that have no DOI cannot currently be managed by this system. This is not a serious limitation because (1) most relevant papers have a DOI and/or a PubMed ID and (2) DOI numbers can be freely generated by third-party services for research documents that have none (e.g., see ResearchGate, 2017).

Unique identification numbers are generated on the fly using the *uuid1()* function of Python's *uuid* package and are attributed to every annotation and parameter instances.

#### Tags

Tags are provided using ontological terms, as discussed in Section Ontologies.

#### Parameters

Modeling parameters are specified as a list of PARAMETER objects (see O'Reilly, 2016b for the format definition) which contains the following elements (described below): a *description*, a list of *required tags*, a *relationship*, and a boolean flag stating whether this parameter is an experimental property (e.g., liquid junction potential, temperature, age of the animals) or not. In the description of these different attributes, we will refer to corresponding examples provided in Table S1, provided in Supplementary Materials. These references will have the following format (Table S1; 1/27–39) to specify the line 27–39 of the example of the first row.

*Required tags* associated with particular types of parameters are defined in the MPCV. They are specified to ensure that a minimal set of information is gathered about annotated parameters, making these annotations more useful for modeling and analyses. For example, the modeling parameter *conductance_ion_curr_max* (i.e., the conductance of the transmembrane ionic flow when all ionic channels related to a particular ionic current are open simultaneously) has the following required tag specification: {“nifext_8054”:“Transmembrane ionic current,” “sao1813327414”:“Cell”}. This means that when the users are annotating values for this type of parameter, they should specify ontological terms for the kind of ionic current and the cell type involved. Both selected terms should be defined in the ontology as children of the “Transmembrane ionic current” and “Cell” terms, respectively (Table S1; 1/27–39). This task is made simple using the NeuroCurator, which automatically populates combo boxes with the available choices.

The *relationship* object is used to specify the entities to which the parameter is related. It can be left undefined, or be specifying a single entity (e.g., an ion current type for a maximal conductance parameter; Table S1; 1/19–23), two entities linked by a directed relationship (e.g., the strength of connectivity from one type of cell to another type of cell; Table S1; 3/26–36) or an undirected relationship (e.g., the correlation of the activity of two brain regions; Table S1; 2/41–51).

Parameter *descriptions* are associated with a specific type of modeling parameter (e.g., the conductance of the leak sodium channels), taken from the list of MPCV terms. They can be defining three types of data: *numerical traces, functions*, and *point values*.

*Numerical traces* are used to specify a set of values for an independent variable (e.g., inactivation time constant) which are associated with values of a dependent variable (e.g., membrane potential) (Table S1; 5/2–37).

The *function* data type is needed to save parameter values when they are obtained by fitting some analytical function to experimental recordings. For example, two parameters (*V*_1/2_ and *k*) are needed to model the steady-state inactivation of a class of ion channels by fitting experimental values to a simple Boltzmann function

$$\begin{array}{l} f=1/\{1+exp\;(V-V_{1/2})/k\} \end{array}$$

where *V* is the membrane potential, *V*_1/2_ is the membrane potential at *f* = 0.5, and *k* is a slope factor (Martina and Jonas, 1997). In such a case, reporting values for *V*_1/2_ and *k* makes sense only if they are associated with the expression of the modeling function *f*. These relationships are preserved by the *function* data type (Table S1; 4/2–28).

*Point values* are used for parameters that are not part of a functional relationship or of a numerical trace (e.g., resting membrane potential; Table S1; 1/3–15).

**Figure 2:**
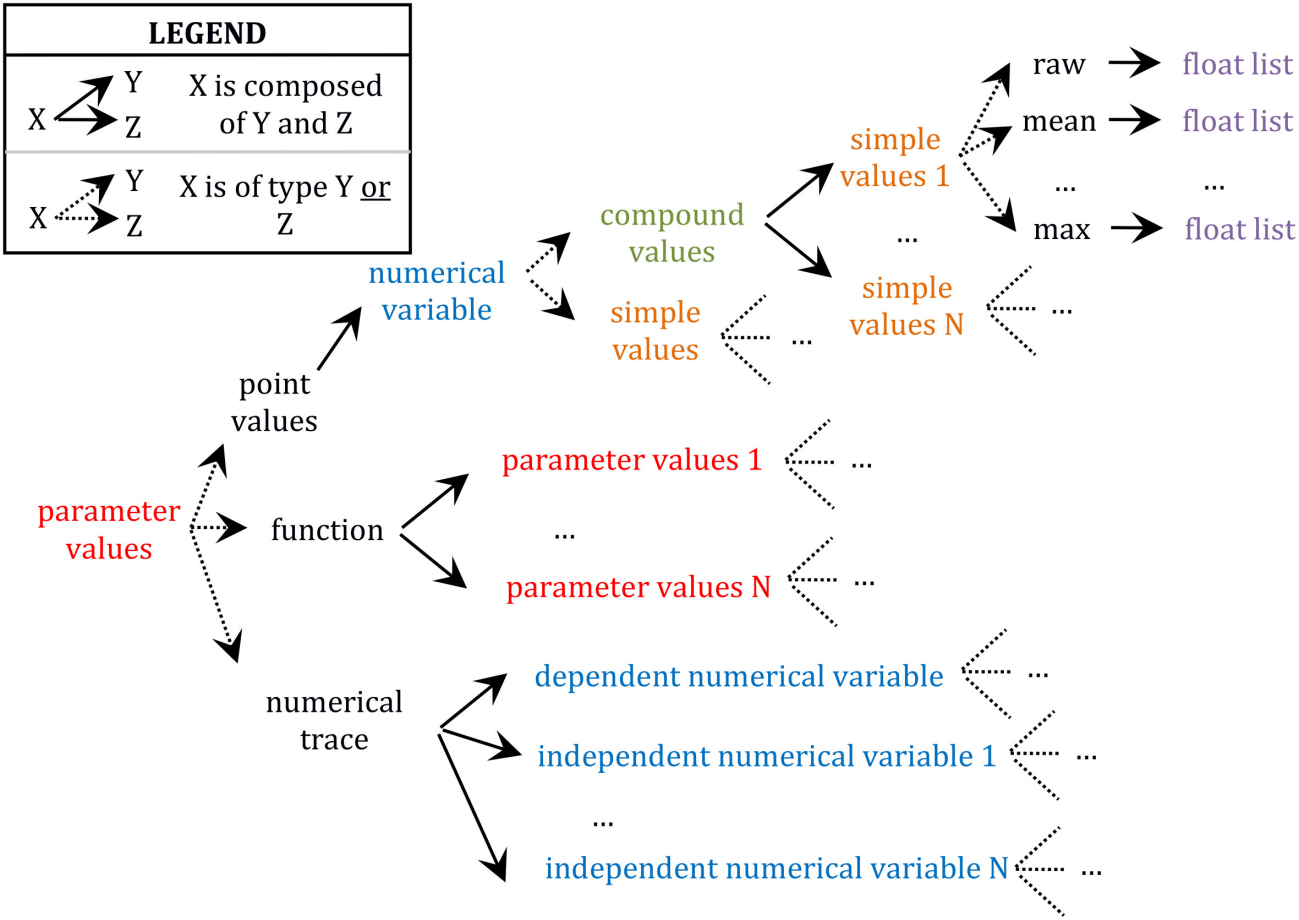
**Schema of the hierarchy of data type encapsulation**. Different types of data are color coded. Only the upper branch of this recursive tree is completely defined. For example, the whole tree starting at “parameter values” would need to be reproduced at “parameter values 1,” the whole tree of “numerical variable” would need to be reproduced at “dependent numerical variable,” etc.

Further, any value encapsulated in these data types (i.e., *numerical trace, function*, and *point value*) can be either specified as a simple value or a compound value. Compound values are aggregates of simple values which are logically related such as X, Y, and Z in “X ± Y (N = Z),” where X is typically a sample mean value, Y its standard error, and Z the size of the sample (e.g., “[…] input resistance (**55** ± **19 M**Ω**;** ***n*** = **94**), resting membrane potential **(**−**60** ± **4 mV;** ***n*** = **67)**, and spike amplitude (**64** ± **7 mV;** ***n*** = **80**) are similar to those of LGN relay neurons […]” in Li et al. (2003); see also Table S1; 2/5–34 for another example of compound values). These X, Y, and Z values must be saved together since they form an interdependent set of statistics such that, for example, Y (a standard error around the mean) is meaningless if reported alone, without X (the mean) and Z (the sample size used to compute the standard error). Finally, simple values can be either “raw” values (i.e., the default category; e.g., the value 5 in “the peak conductance density for the non-inactivating K^+^ current was chosen to be 5 pS/μm^2^” (Haeusler and Maass, 2006) is a “raw” value in the sense that it is not a statistic computed from a sample) or some statistics (e.g., mean, median, standard deviation, maximum, etc.; see also Table S1; 2/8–12 for an example of a annotation of a mean value) and they are always specified as lists of floating-point values with one or more items depending on the availability of single or repeated measures. This complex hierarchical encapsulation of value types is illustrated in Figure 2.

Annotated values should be identical to published numbers and should not be transformed in any ways. Values are saved alongside with their unit (as specified in the paper) and are checked for consistency using Python's *quantities* package.

#### Experimental properties

Annotated parameters can be marked as experimental properties so that they can be associated with other annotations to specify the experimental context. For example, an annotation defining a “slice_thickness” parameter can be associated as an experimental property of a second annotation specifying a “neuron_density” parameter in mm^−2^ (e.g., evaluated with the dissector method). Such an association allows, before integrating this density to a model, to convert its value from a surface density (published value) to a volume density (value needed for modeling) by dividing the annotated “neuron_density” per the “slice_thickness” used for the counting procedure.

Note, that two different types of information define the complete experimental context: categorical (through tagging; e.g., “Wister rat”) and numerical (through annotated parameters marked as experimental properties; e.g., age = 14 days).

#### Localizer

Various ways to localize annotations are provided to account for the different use cases. A *text* type of annotation is defined by a segment of text and the exact position (specified as the character number) where it starts in the curated document. To provide an unambiguous localization, the publication PDF is first parsed to generate a corresponding plain-text file, which is kept as a reference (see Supplementary Materials Section Verification of publication access rights for details). To preserve the reliability of annotation localization, once created, this file should never be changed or replaced. For that reason, this localization key is saved centrally on a server (see Supplementary Documents Materials Avoiding Copyright Issues for copyright issues related with sharing this key with clients).

In general, if the information to annotate is contained in a figure, a table, or an equation, these can be entered using the respective annotation type and specifying the respective number. These numbers are encoded as strings rather than integers to allow more flexibility for the different use cases (e.g., “1,” “1.c,” “III,” “from 4 to 10,” “1, upper-left panel”). For tables, the user can also specify a row and a column number, if those are not ambiguous (i.e., row and column numbers are not ambiguous when the shape of the table is such that it can be represented as a matrix). Finally, the *position* type is provided for situations where all other types are not appropriate. This can be the case, e.g., if the curator wants to annotate a very specific portion of a figure. These annotations are specified by the number of the page and the coordinate of a bounding box encompassing the content to be annotated. These are specified on the reference PDF file^3^. These are freely accessible for open access publications. For copyrighted material, access is granted only to users who have demonstrated that they already own a copy of the paper.

### Zotero library

The proposed architecture integrates a citation database synchronized with a Zotero library (http://www.zotero.org). This citation management software has been chosen because it is free, cross-platform, and open-source. It is maintained by a University center (Roy Rosenzweig Center for History and New Media from George Mason University) and it offers a convenient Python API. Using Zotero allows integrating this framework more naturally with the existing software environment and avoid creating custom solutions for features already well covered by existing software. For the creation of our corpus, collaborative curation work was promoted by synching with a group library.

### Global software infrastructure

**Figure 3:**
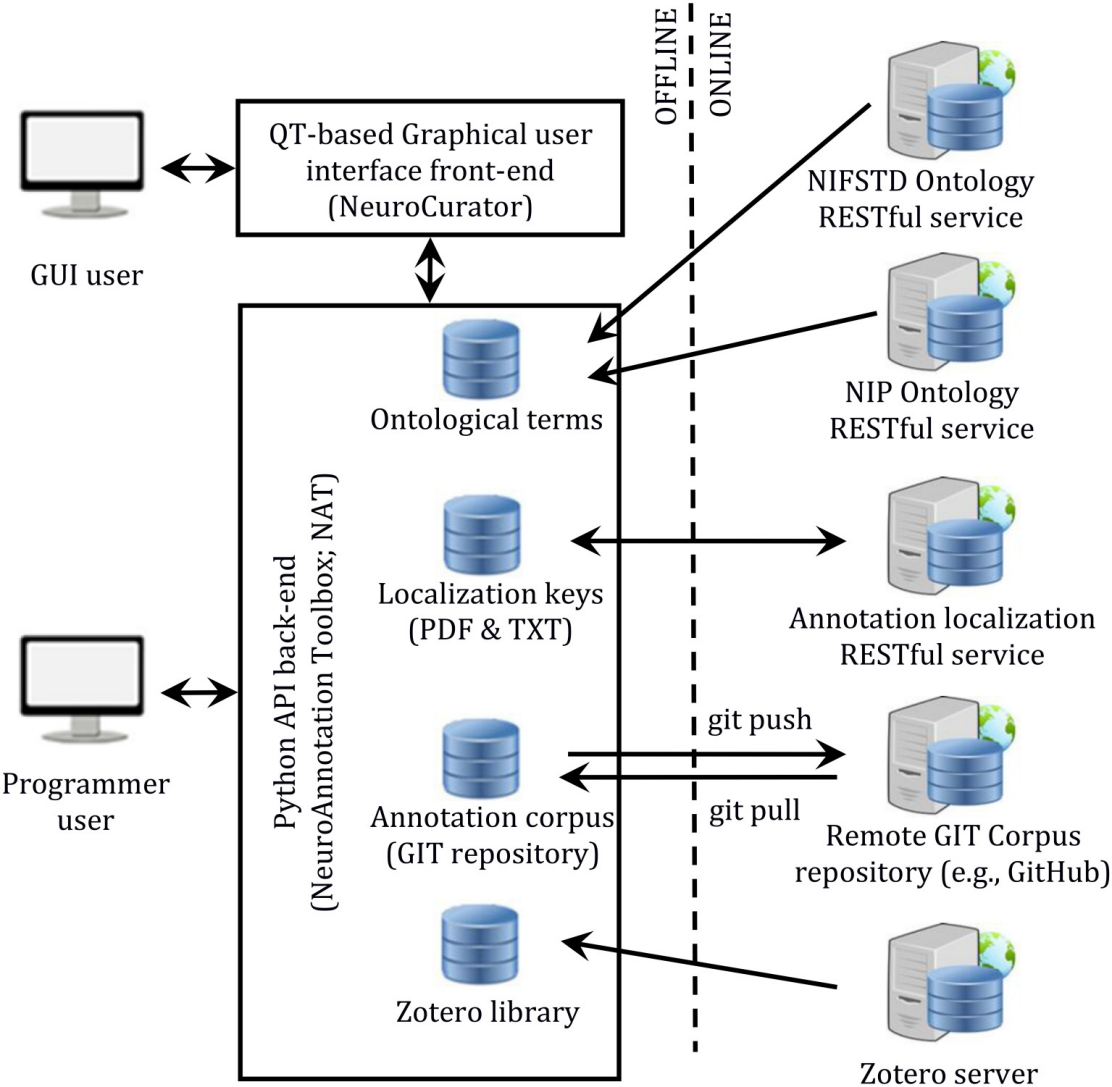
**Software infrastructure proposed for literature curation**. Constitution and consultation of annotation corpora can be made either through a user-friendly graphical interface (NeuroCurator) or programmatically in Python using the NAT package. Both the NeuroCurator and the NAT package can be used offline. They require connectivity only when new resources (i.e., resources not already stored locally) are needed or for synching the local GIT version of the corpus with a remote one.

The complete system is composed of a few components: a front-end (NeuroCurator), a back-end (NeuroAnnotation Toolbox; NAT), a RESTful service for managing localization keys, RESTful ontology services (NIFSTD and NIP), the Zotero server for centralizing the citation library, and a GIT server for versioning the annotation corpus. This architecture is depicted in Figure 3.

### User interface

A graphical user interface (GUI) named NeuroCurator has been created as a front-end to provide all the functionalities required for a flexible and efficient curation process. It is coded using *PySide* (Python bindings for *QT*, a cross-platform C++ toolbox for creating GUIs). It allows displaying the publications contained in the Zotero library (Figure S1), to create new annotations, visualize or modify existing ones, associate tags to annotations by selecting ontological terms from those stored locally or by searching the ontologies online (Figure S2), to annotate new modeling parameters (Figure S3), and to search for annotations in the corpus according to flexible user-defined queries (Figure S4). The development of the NeuroCurator is an ongoing project and the main objective is to provide an efficient and enjoyable user experience to stimulate the adoption of this framework by the community.

The code of the NeuroCurator has been separated from the back-end, which constitute a Python package named NeuroAnnotation Toolbox (NAT). This separation allows interacting with annotation corpora programmatically (e.g., from an IPython Notebook) without having to install the NeuroCurator and its dependencies.

The front-end supports creating annotations using the full expressiveness of the annotation format described previously. Specifically, concerning the localization of the annotations, a citation can be localized by pasting a snippet of text and clicking on the “Localize” button. This action initiate a search for corresponding text and proposes options to the user if more than one similar text is found in the document. For localization according to position in the PDF, the interface allows the user to specify the region of interest by drawing a bounding box over any page of the PDF. For the other types of annotations, the user has to specify it as plain text (i.e., number of the figure, table or equation). The graphical interface does not allow yet to visualized annotated information overlaid on the PDF. Implementation of such a functionality using a third-party PDF viewer is considered for future work.

## Case study: corpus of annotation for the modeling of the thalamo-cortical loop

### The corpus

An example of annotation corpus is already available on GitHub (see Table 1). At the moment of writing, this corpus was containing 435 manually made annotations and 257 annotated parameters from 80 different publications. This corpus is centered on the biologically detailed modeling of the thalamo-cortical loop for the somatosensory cortex of the rat. It is an ongoing curation task in the context of the Blue Brain Project. A histogram showing the number of annotated parameters for the 30 most annotated parameter types is shown on Figure 4. A Jupyter notebook has been included to the code base of the NAT project, which allows to compute and show an up-to-date version of these information (https://github.com/BlueBrain/nat/blob/master/notebooks/Status_thalamus_corpus.ipynb).

**Table 1:** **List of key open-access resources constituting the annotation framework**.

	**Resource**	**Location**
1	NeuroAnnotation Toolbox	https://github.com/BlueBrain/nat
2	NeuroCurator application	https://github.com/BlueBrain/neurocurator
3	Thalamo-cortical loop annotation corpus	https://github.com/BlueBrain/corpus-thalamus
4	REST end-point for annotation localization^#^	https://bbpteam.epfl.ch/
5	Documentation of the REST API for the NIP ontology^*^	https://collab.humanbrainproject.eu/#/collab/47/nav/7267
6	REST end-point for the NIP ontology^*^,^#^	https://nip.humanbrainproject.eu/api/scigraph/
7	Documentation of the REST API for the NIFSTD ontology^*^	http://trinity.neuinfo.org:9000/scigraph/docs/
8	REST end-point for the NIFSTD ontology^*^,^#^	http://trinity.neuinfo.org:9000/scigraph/

* *These resources are not under the responsibility of the authors, but are used as external services by the infrastructure*.

# These resources are the root of the REST API end-point. Thus, they cannot be directly opened as a web page. A sub-command must be added (e.g., http://trinity.neuinfo.org:9000/scigraph/graph/properties)

**Figure 4:**
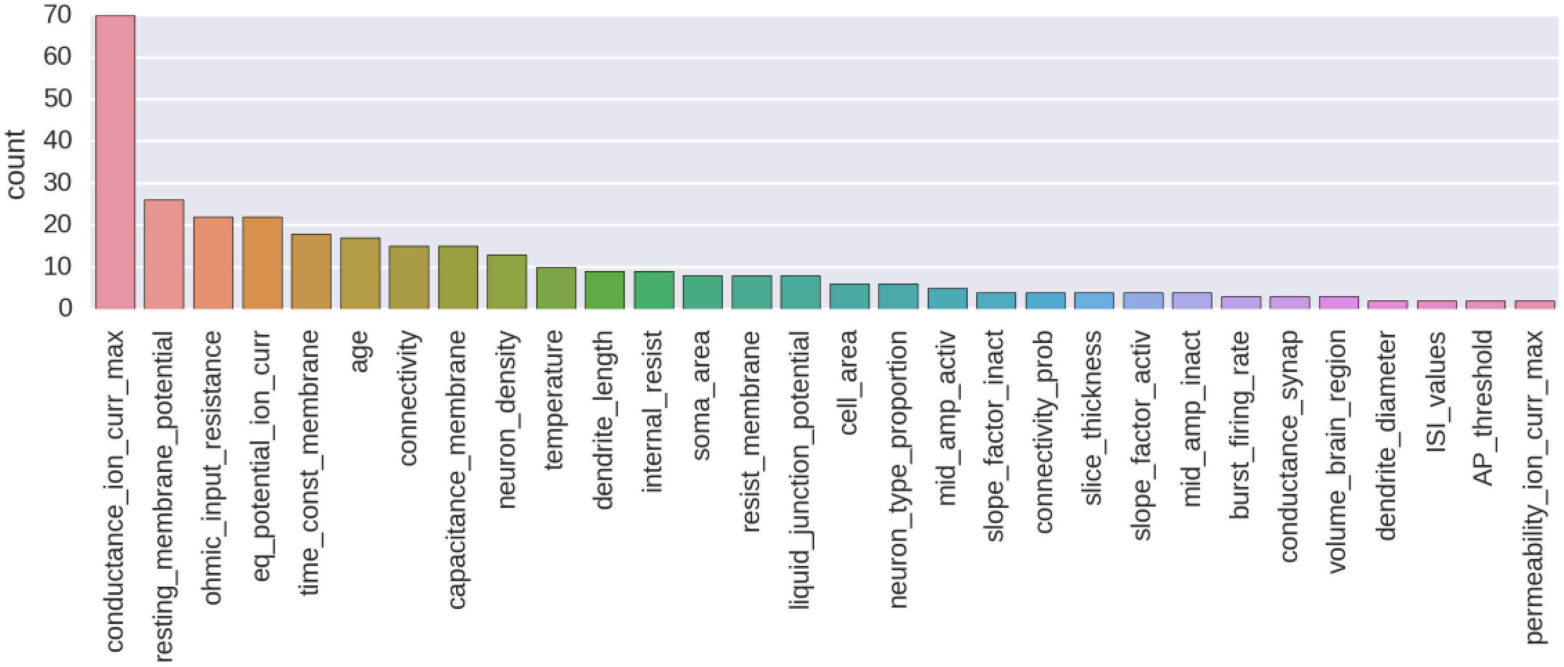
**Histogram showing the number of annotated parameters for the 30 most annotated parameter types**.

### Interaction with the corpus

#### First example

**Figure 5:**
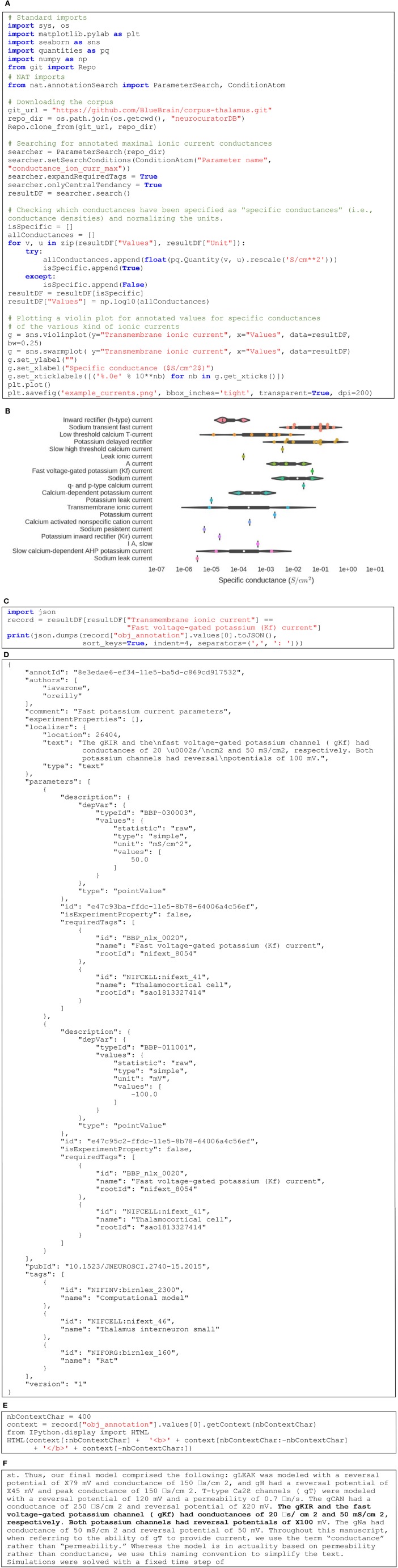
**(A)** Python code to query the corpus and plotting maximal conductances for various ionic currents. **(B)** Resulting set of violin plots showing the distribution of maximal conductances of ionic currents annotated in the corpus. **(C)** Querying for the annotation of a specific point in the plot. **(D)** JSON representation of the corresponding annotation. **(E)** Query to get the annotated text within its context. **(F)** Localized text in its context (in this case, 400 characters before and after the annotated text).

Figure 5 gives an example of how NAT can be used to interact programmatically with a corpus. Figure 5A first shows how to get a local copy of our sample corpus by performing a “git clone” operation through Python. Then, it describes how to search for values of a specific parameter and visualize the corresponding data. In this example, we are querying for all annotated values of maximal ionic conductance and plot those that are defined as *specific* conductance only (i.e., conductance normalized by area of cell membrane). The Figure 5B illustrates the resulting violin plot, separating annotated values per type of ionic currents. The code in Figure 5C shows how to get a specific annotation and print its JSON representation (see Figure 5D for the output). Finally, the code in Figure 5E demonstrates how to display this annotated content in its context. In this case, it is a *text* annotation so its context is defined by the surrounding text. Figure 5F shows the output: the annotated text displayed in bold, surrounded by the 400 preceding and following characters. The complete Jupyter notebook reproducing this example can be consulted online (O'Reilly, 2016c).

#### Second example

**Figure 6:**
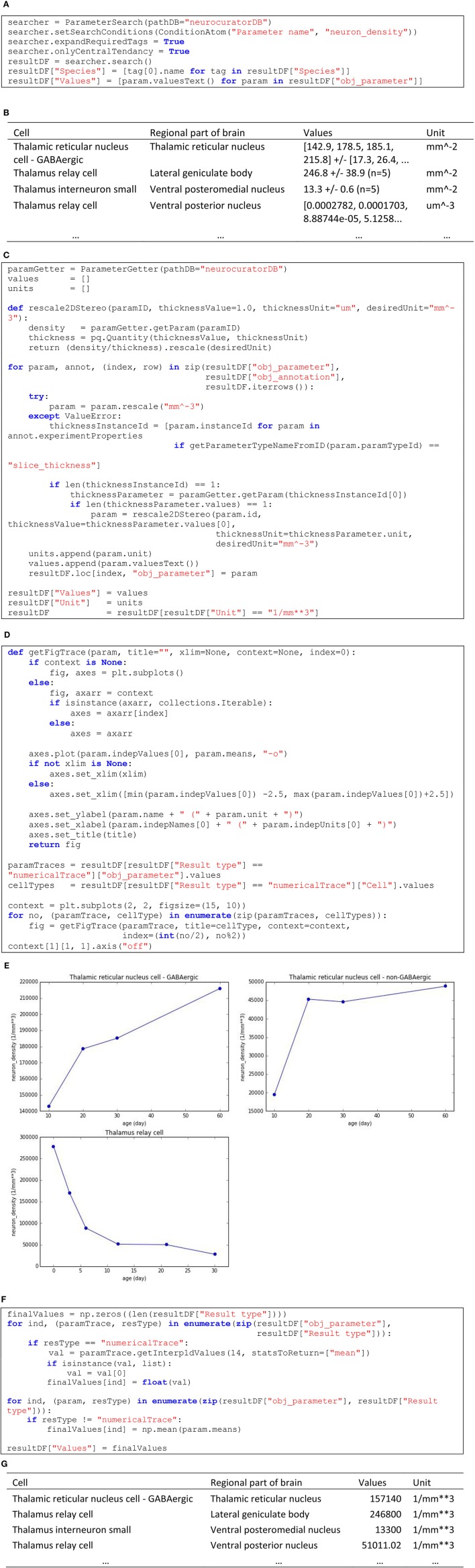
**(A)** Code to list annotated neuronal densities. **(B)** Extract of the resulting table from **(A)**. **(C)** Code to rescale to mm^−3^ unit (applying 2D to 3D transformation using slice thickness whenever appropriate) and to keep only the annotations that could successfully be rescaled. **(D)** Code to display the three annotations that are specified as numerical traces. **(E)** Resulting plots from **(D)**. **(F)** Code for keeping only the values interpolated at 14 days old for numerical traces and only the mean for compound values. **(G)** Resulting table from **(F)**, which displays homogeneous values and units.

In this second example, we are interested in collecting all the information about neuron densities annotated from stereological studies and express them in a homogeneous format so that they can be integrated in a modeling processes. Skipping corpus download and package imports (see O'Reilly, 2016d for the complete and executable notebook related to this example), Figure 6A shows how to query the corpus to obtain the values for the “neuron_density” parameter and Figure 6B shows an extract of the *resultDF* table. As can be seen in this tables, units are not homogeneous (mm^−2^ and um^−3^). Figure 6C shows how these units can be normalized, using when necessary the annotated slice thickness to transform from area to volume. In the same table, we can see that the values are also specified in a heterogeneous way. The first and fourth rows of the extract show two annotations that are actually numerical traces. In total, in the current corpus, there are three such annotations of cell densities. Corresponding numerical traces can be plotted as shown in Figure 6D (code) and Figure 6E (resulting plots). Also, other parameters specify compound values (row 2 and 3 in Figure 6B) as mean ± standard error (*N* = sample size). To homogenize these different value formats, we interpolate numerical traces to obtain densities at 14 days old (supposing that this is the age of the rat brain we want to model) and take only the mean of compound values (see code in Figure 6F). The resulting table (see Figure 6G for an extract) now contains parameters that are homogeneous in units and values, making them appropriate for integration into a model of a rat^4^ thalamus with different cell types and brain regions.

## Availability

This project aims at promoting collaborative literature curation and reproducible integration of literature information into neuronal modeling pipelines and analyses. Accordingly, the resources described in this paper are all open-access. Table 1 list location of the different resources.

## Discussion

### A better literature curation for a more integrated knowledge in neuroscience

The study of neuroscience is challenged by the extreme complexity of the brain functioning, the broad spectrum of expertise required to pull together all the evidences from different fields, and the wide range of scales involved in understanding the mechanisms at play. This often results in different research threads being performed *in silo* (i.e., in parallel, without synergy), with too little cross-scale and cross-discipline integration of the knowledge. At the same time, all the efforts invested in understanding how the brain works have resulted in an explosion of both the amount of experimental data produced and the size of the published literature. This can be seen as a curse if no infrastructure is put in place to manage this big data, or it can be turned into a blessing if *a contrario* tools and methodologies are adopted to integrate this knowledge synergistically. To contribute into this direction, we have developed a framework that supports collaborative curation of literature so that corpora of relevant facts and experimental values can be built and shared across brain modeling projects. All the tools developed in this project are open-access. They are and will continue to be in heavy development as they are part of the large-scale modeling endeavor being conducted within the Blue Brain Project. Contributions from the community in the form of feedback, constructive criticism, code patches or extensions are most welcome.

### Limitations

There is a real challenge in developing a literature annotation framework that captures the different kind of data published in the literature, and yet to provide some means to homogenize them in a format that is usable in modeling, without losing traceability. In the development of this annotation framework, the focus has been placed on capturing faithfully the variability. There is still a need for developing a more comprehensive set of routines for homogenizing the annotated data into a consumable form for the different modeling requirements.

The current framework also misses a systematic support for cross-referencing between publications, for example to capture in a formal way (as opposed to a free-form comment) the normalization of a parameter annotated in one paper by a factor annotated in a second paper.

Finally, ontologies have been embedded in this annotation framework mainly as controlled vocabularies used for systematic tagging. Semantically richer possibilities (e.g., adding more complex ontological constructs involving relationships between entities of an annotated text) was out of our scope. On a related topic, the MPCV created for this work could arguably be improved and made into a stand-alone ontology development project following OBO Foundry's principles.

### Future directions

This paper is the first of a two-paper series. In the second paper, we will discuss how created corpora can be integrated into modeling workflows to support a reproducible and traceable use of literature information. It is also in our future goals to better connect this framework with existing tools, either by interfacing them as producers (i.e., integrating annotations made by other software such text-mining applications or with different annotation interfaces such as https://hypothes.is) or as consumer (i.e., publishing curated information in third-party portals such as knowledge-space.org). The resources necessary to address the limitations described previously will be invested depending on the evolution of the needs expressed by the neuroscientific community.

## Author contributions

CO has programmed the software and wrote the first draft of the paper. CO and EI have made the annotation corpus. EI has provided user feedback, bug reports, feature request. EI and SH have revised and edited the manuscript. SH has provided supervision for this project.

## Funding

This project has been founded by the EPFL Blue Brain Project Fund, the ETH Board Funding to the Blue Brain Project, and through OpenMinted (Grant 654021), a H2020 project funded by the European Commission.

### Conflict of interest statement

The authors declare that the research was conducted in the absence of any commercial or financial relationships that could be construed as a potential conflict of interest.

## Footnotes

^1^This project currently curates 47 electrophysiological properties. Correspondences between NeuroElectro and MPCV terms have been made explicit in a CSV file (modelingDictionary_relationships.csv) part of the NAT code base, pending the restructuration of MPCV in a more formal ontology.

^2^Pretty-printing and fixed JSON element ordering are not necessary for syntactic validity of the annotation files but allows for better human-readability and, most importantly, it makes the use of GIT to track modifications or resolve merging conflicts much more practical.

^3^Although PDF versions of publications are fairly consistent, some sources add for example front pages. Alternative versions may also include Supplementary documents or not. Different scanning of a same paper may have different alignments due, for example, to different page format at scanning time. For all these reasons, it is important to provide a consistent reference version of publication PDFs.

^4^Not shown here is the fact that all these annotations are associated with “rat” ontological terms.

## References

[B1] Bada M. Eckert M. Evans D. Garcia K. Shipley K. Sitnikov D.. (2012). Concept annotation in the CRAFT corpus. BMC Bioinformatics 13:161. 10.1186/1471-2105-13-161 22776079 PMC3476437

[B2] EPFL (2017). Blue Brain Project - EPFL. Available online at: http://bluebrain.epfl.ch/ (Accessed April 4, 2017).

[B3] French L. Liu P. Marais O. Koreman T. Tseng L. Lai A.. (2015). Text mining for neuroanatomy using WhiteText with an updated corpus and a new web application. Front. Neuroinform. 9:13. 10.3389/fninf.2015.00013 26052282 PMC4439553

[B4] Ghazvinian A. Noy N. F. Musen M. A. (2011). How orthogonal are the OBO Foundry ontologies? J. Biomed. Semant. 2(Suppl. 2):S2. 10.1186/2041-1480-2-S2-S2 21624157 PMC3102891

[B5] Haeusler S. Maass W. (2006). A statistical analysis of information-processing properties of lamina-specific cortical microcircuit models. Cereb. Cortex 17, 149–162. 10.1093/cercor/bhj132 16481565

[B6] Imam F. T. Larson S. D. Bandrowski A. Grethe J. S. Gupta A. Martone M. E. (2012). Development and use of ontologies inside the neuroscience information framework: a practical approach. Front. Genet. 3:111. 10.3389/fgene.2012.00111 22737162 PMC3381282

[B7] Kim J.-D. Ohta T. Tateisi Y. Tsujii J. (2003). GENIA corpus—a semantically annotated corpus for bio-text mining. Bioinformatics 19(Suppl. 1), i180–i182. 10.1093/bioinformatics/btg1023 12855455

[B8] Larson S. D. Martone M. E. (2013). NeuroLex.org: an online framework for neuroscience knowledge. Front. Neuroinform. 7:18. 10.3389/fninf.2013.00018 24009581 PMC3757470

[B9] Le Franc Y. Davison A. P. Gleeson P. Imam F. T. Kriener B. Larson S. D. (2012). Computational neuroscience ontology: a new tool to provide semantic meaning to your models. BMC Neurosci. 13:P149 10.1186/1471-2202-13-S1-P149

[B10] Li J. Bickford M. E. Guido W. (2003). Distinct firing properties of higher order thalamic relay neurons. J. Neurophysiol. 90, 291–299. 10.1152/jn.01163.2002 12634282

[B11] Markram H. Muller E. Ramaswamy S. Reimann M. W. Abdellah M. Sanchez C. A.. (2015). Reconstruction and simulation of neocortical microcircuitry. Cell 163, 456–492. 10.1016/j.cell.2015.09.029 26451489

[B12] Martina M. Jonas P. (1997). Functional differences in Na+ channel gating between fast-spiking interneurones and principal neurones of rat hippocampus. J. Physiol. 505(Pt 3), 593–603. 9457638 10.1111/j.1469-7793.1997.593ba.x PMC1160038

[B13] NCBI (2017). PubMed. Available from: http://www.ncbi.nlm.nih.gov/pubmed (Accessed April 4, 2017).

[B14] O'Reilly C. (2016a). Introduction to the NeuroInformatics Platform - FENS 2016 - Part 3 - Ontology Services. Available online at: https://github.com/christian-oreilly/NIP_FENS2016/blob/master/Part3-Ontologies.ipynb (Accessed August 30, 2016).

[B15] O'Reilly C. (2016b). NeuroAnnotation Toolbox Wiki - Annotation Format. Available online at: https://github.com/BlueBrain/nat/wiki/Annotation-format (Accessed August 30, 2016).

[B16] O'Reilly C. (2016c). Example of Interaction with a Corpus Using the NeuroAnnotation Toolbox - Ionic Currents. Available online at: https://github.com/BlueBrain/nat/blob/master/notebooks/Example%20ionic%20currents.ipynb (Accessed August 30, 2016).

[B17] O'Reilly C. (2016d). Example of Interaction with a Corpus Using the NeuroAnnotation Toolbox - Stereology. Available from: https://github.com/BlueBrain/nat/blob/master/notebooks/ThalamusStereology.ipynb. (Accessed September 16, 2016).

[B18] Ozyurt I. B. Grethe J. S. Martone M. E. Bandrowski A. E. (2016). Resource disambiguator for the web: extracting biomedical resources and their citations from the scientific literature. PLoS ONE 11:e0146300. 10.1371/journal.pone.0146300 26730820 PMC5156472

[B19] Perkel J. M. (2015). Annotating the scholarly web. Nature 528, 153–154. 10.1038/528153a 26632591

[B20] Richardet R. Chappelier J. C. Tripathy S. Hill S. (2015). Agile text mining with Sherlok, in Proceedings of the IEEE International Conference on Big Data (Santa Clara), 1479–1484.

[B21] ResearchGate (2017). Generating a DOI. Available from: https://explore.researchgate.net/display/support/Generating+a+DOI (Accessed April 4, 2017).

[B22] Smith B. Ashburner M. Rosse C. Bard J. Bug W. Ceusters W.. (2007). The OBO Foundry: coordinated evolution of ontologies to support biomedical data integration. Nat. Biotechnol. 25, 1251–1255. 10.1038/nbt1346 17989687 PMC2814061

[B23] Stenetorp P. Pyysalo S. Topić G. Ohta T. Ananiadou S. Tsujii J. (2012). BRAT: A Web-based Tool for NLP-assisted Text Annotation, in: Proceedings of the 13th Conference of the European Chapter of the Association for Computational Linguistics (Avignon), 102–107.

[B24] Thompson H. S. McKelvie D. (1997). Hyperlink semantics for standoff markup of read-only documents, in Proceedings of SGML Europe' 97: The Next Decade – Pushing The Envelope (Barcelona), 227–229.

[B25] Tripathy S. J. Savitskaya J. Burton S. D. Urban N. N. Gerkin R. C. (2014). NeuroElectro: a window to the world's neuron electrophysiology data. Front. Neuroinform. 8:40. 10.3389/fninf.2014.00040 24808858 PMC4010726

[B26] Yimam S. M. Gurevych I. (2013). WebAnno: A flexible, web-based and visually supported system for distributed annotations, in Proceedings of the 51th Annual Meeting of the Association for Computational Linguistics (ACL) - System Demonstrations (Sofia), 1–6.

