# Supplementary material for: A Framework for Collaborative Curation of Neuroscientific Literature
Source: Front Neuroinform. 2017 Apr 19;11:27. doi: 10.3389/fninf.2017.00027 (PMC5395614; doi:10.3389/fninf.2017.00027)
Supplement: Supplementary file 1 [file DataSheet1.pdf]

Supplementary Material

A Framework for Collaborative Curation of Neuroscientific Literature

Christian O`Reilly *, Elisabetta Iavarone and Sean Hill

*** Correspondence:** Corresponding Author: christian.oreilly@epfl.ch

1 Technical details

1.1. Integration of NIPSTD and NIP ontologies

Integrating the NIFSTD and NIP ontologies to our system involved some technical difficulties. These ontologies include a very large number of terms, most of which will probably never be used in annotations. From one point of view, this large coverage is desirable to provide a flexible annotation process and to avoid constraining the system usability by our current views of its future potential. However, this overabundance makes integrating these ontologies directly to the software code not practical because they would significantly increase the size of the project. Further, it would require implementing specifically dedicated and optimized ontology search algorithms to avoid running into performance issues when using features such as live auto-completion for tag entry.

Alternatively, ontologies can be queried through their online REST API. However, this approach also induces some inefficiencies (mostly because of network communication delays) and, most importantly, it makes the use of the tool dependent on the availability of an Internet connection, which is an important limitation to its usability.

The tradeoff adopted in our design is to store locally all the terms previously used. Thus, after a minimal amount of work with the system, terms needed for most annotation cases are available offline. Yet, the flexibility offered by a large ontological coverage is not compromised since unused terms can still be fetched online when needed.

1.2. Avoiding copyright issues

As previously described, depending on the context, two different publication file formats are used to localize annotations: PDF and plain text. Identical versions of these reference files need to be shared across all users to provide reliable localization. This must be implemented as a centralized service offered within the annotation framework. External sources can provide publications in different supports (e.g., HTML, PDF, XML, plain text) or different versions (e.g., added front-pages, enriched PDF versions, different optical character recognition), which makes these documents unreliable for localization across users. However, because of copyrights, many reference files cannot be freely distributed to users who don't already own a copy of the publication. To solve this issue, we have designed a RESTful service that allows reliable localization without infringing copyrights.

This service is used to import new publications to the server, to get a local copy of the server-side PDF and text versions, and to visualize annotations in their contexts.

Obviously, accessing the features provided by the RESTful service requires Internet access. To minimize this dependency, once the access rights of a user to a publication has been verified (see section 1.3), corresponding server-side files are saved locally so that no network connectivity is required anymore to work on that publication.

1.3. Verification of publication access rights

When a publication PDF is imported to the server, it is first parsed to create a plain-text version using the *pdftotext* command-line program. If the resulting document is less than 2 KB, it contains virtually no text. Likely, such a PDF is a scanned version on which no optical character recognition (OCR) has been performed. In that case, OCR is performed on the document by the server using the *Tesseract Open Source OCR Engine*.

Users have to send their own copy of the publication PDF to be able to download from the server the corresponding reference documents (PDF and text). When the server receives a user PDF, it computes its MD5 hash and compares it to MD5 hashes previously associated with this publication. If a match is found, the server-side documents are sent to the client to serve as localization keys. Otherwise, the user PDF is converted to a text version using the same process previously described for importing publications to the server. The generated text file is compared against the reference version and an index of similarity is computed between both texts. If this similarity index is high enough, the MD5 hash from the user PDF is associated with the corresponding publication and the server-side versions of the PDF and text files are sent to the client. If the similarity index is too low, the client is denied access to the requested server files since his/her right to access this publication could not be established. The concerned researcher can nevertheless still use the RESTful service to visualize annotations in their context since sharing small extract of copyrighted material generally present no legal issues.

This two steps procedure (i.e., first checking the MD5, then checking text similarity) has been implemented mainly to minimize the need to perform lengthy OCR on users' PDFs. Also, using only MD5 verification would not be sufficient since any modification to the PDF (e.g., adding a "sticky note" comment) would change its MD5 hash and result in access denial.

2 Supplementary Tables

Table S1. Example of parameter annotations.

Row	Annotation in its context	JSON representation	Line
1	[...] were modeled with a reversal potential of 120 mV and a permeability of 0.7 μm/s. **The g_{CAN} had a conductance of 250 μS/cm^{2}** and reversal potential of -20 mV. The g_{KIR} and the fast voltage-gated potassium channel (g_{Kf}) [...] (1)	"parameters": [<br>{<br>"description": {<br>"depVar": {<br>"typeId": "BBP-030003",<br>"values": {<br>"statistic": "raw",<br>"type": "simple",<br>"unit": "uS/cm^2",<br>"values": [<br>250.0<br>]<br>}<br>},<br>"type": "pointValue"<br>},<br>"id": "e47c87ee-ffdc-11e5-8b78-64006a4c56ef",<br>"isExperimentProperty": false,<br>"relationship": {<br>"entity1": {<br>"id": "NIFCELL:nifext_41",<br>"name": "Thalamocortical cell"<br>},<br>"entity2": "None",<br>"type": "point"<br>},<br>"requiredTags": [<br>{<br>"id": "BBP_nlx_0016",<br>"name": "Calcium activated nonspecific<br>cation current",<br>"rootId": "nifext_8054"<br>},<br>{<br>"id": "NIFCELL:nifext_41",<br>"name": "Thalamocortical cell",<br>"rootId": "sao1813327414"<br>}]}]	1<br>2<br>3<br>4<br>5<br>6<br>7<br>8<br>9<br>10<br>11<br>12<br>13<br>14<br>15<br>16<br>17<br>18<br>19<br>20<br>21<br>22<br>23<br>24<br>25<br>26<br>27<br>28<br>29<br>30<br>31<br>32<br>33<br>34<br>35<br>36<br>37<br>38
2	[...] and 33 TRN neurons from WT mice were injected with NB using the injection method described above.	"parameters": [<br>{ "description": {<br>"depVar": {<br>"typeId": "BBP-110005",<br>"values": {<br>"type": "compound",<br>"valueLst": [<br>{"statistic": "mean",	1<br>2<br>3<br>4<br>5<br>6<br>7<br>8

	**The mean number of neurons per cluster (including the dye-injected neuron) was 9 +/- 2.5 neurons (range, 1–24) from rat** and 8.7 +/- 0.9 neurons (range, 2–21) from mouse (Fig. 2A). In the mouse TRN, injections were performed [...] (2)	"type": "simple",<br>"unit": "dimensionless",<br>"values": [9.0]<br>},<br>{ "statistic": "sem",<br>"type": "simple",<br>"unit": "dimensionless",<br>"values": [2.5]<br>},<br>{ "statistic": "N",<br>"type": "simple",<br>"unit": "dimensionless",<br>"values": [9.0]<br>},<br>{ "statistic": "min",<br>"type": "simple",<br>"unit": "dimensionless",<br>"values": [1.0]<br>},<br>{ "statistic": "max",<br>"type": "simple",<br>"unit": "dimensionless",<br>"values": [24.0]<br>}<br>]<br>}<br>},<br>"type": "pointValue"<br>},<br>"id": "7eeb0b54-88b6-11e6-b01f-64006a4c56ef",<br>"isExperimentProperty": false,<br>"relationship": {<br>"entity1": {<br>"id": "NIFCELL:nifext_45",<br>"name": "Thalamic reticular nucleus cell"<br>},<br>"entity2": {<br>"id": "NIFCELL:nifext_45",<br>"name": "Thalamic reticular nucleus cell"<br>},<br>"type": "undirected "<br>},<br>"requiredTags": [<br>{ "id": "NIFCELL:nifext_45",<br>"name": "Thalamic reticular nucleus cell",<br>"rootId": "NIFCELL:sao1813327414"<br>},<br>{ "id": "NIFSUB:sao1311109124",<br>"name": "Electrotonic Synapse",<br>"rootId": "NIFSUB:sao914572699"<br>}]}]
3	[...] This figure shows that the S1 cortex was	"parameters": [<br>{ "description": {<br>"depVar": {

	the largest projection target in 4 of 5 anterior POm neurons and 4 of 5 posterior POm neurons. **In the S1 cortex, 6.1-38.5% (mean ± SD = 20.6 ± 14.6%) of axon varicosities of anterior POm neurons 2-5 were distributed in L1**, whereas 40.0-91.7% (64.1 ± 19.4%) of varicosities of posterior POm neurons 6-10 were in L1. This difference was highly [...] (3)	"typeId": "BBP-110001",<br>"values": {<br>"type": "compound",<br>"valueLst": [<br>{ "statistic": "mean",<br>"type": "simple",<br>"unit": "dimensionless",<br>"values": [0.206]<br>},<br>{ "statistic": "sd",<br>"type": "simple",<br>"unit": "dimensionless",<br>"values": [0.146]<br>}<br>]<br>}<br>},<br>"type": "pointValue"<br>},<br>"id": "8432d9a6-009a-11e6-9d99-c869cd917532",<br>"isExperimentProperty": false,<br>"relationship": {<br>"entity1": {<br>"id": "NIFGA:birnlex_939",<br>"name": "Posterior nucleus of thalamus"<br>},<br>"entity2": {<br>"id": "NIFGA:nlx_anat_090807",<br>"name": "Neocortex layer 1"<br>},<br>"type": "directed"<br>},<br>"requiredTags": []<br>}]}]
4	[...] The steady-state inactivation (h∞) curve was fitted with a simple Boltzmann function: **f = 1/{1 + exp[(V – V½)/k]}.** All numerical values given denote means ± s.e.m. [...] (4)<br><br>[Note: In this case, this annotation was made as a text rather than an equation	"parameters": [<br>{ "description": {<br>"depVar": {<br>"statistic": "raw",<br>"typeId": "BBP-050002",<br>"unit": "dimensionless"<br>},<br>"equation": "prop_inact_ion_curr = 1/(1+exp((potential_membrane - mid_amp_inact)/slope_factor_inact))",<br>"indepVars": [<br>{ "statistic": "raw",<br>"typeId": "BBP-010001",<br>"unit": "mV"<br>}<br>],<br>"parameterRefs": [<br>{ "instanceId": "762f45f0-eade-11e5-94e2-64006a4c56ef",<br>"paramTypeId": "BBP-090002"

	because there was no number associated to this equation in the text]	},<br>{ "instanceId": "7f3542e0-eb82-11e5-ac32-64006a4c56ef",<br>"paramTypeId": "BBP-101002"<br>}<br>],<br>"type": "function"<br>},<br>"id": "71233092-eb80-11e5-a9b7-64006a4c56ef",<br>"isExperimentProperty": false,<br>"relationship": {<br>"entity1": {<br>"id": "NIFMOL:nifext_8055",<br>"name": "Sodium current"<br>},<br>"entity2": "None",<br>"type": "point"<br>},<br>"requiredTags": [<br>{ "id": "NIFMOL:nifext_8054",<br>"name": "Transmembrane ionic current",<br>"rootId": "nifext_8054"<br>},<br>{ "id": "NIFCELL:sao1813327414",<br>"name": "Cell",<br>"rootId": "sao1813327414"<br>}]}]
5	Table 1<br>Row: 1, 3, 5, 7<br>Column: 1<br>(5)	"parameters": [<br>{"description": {<br>"depVar": {<br>"typeId": "BBP-131001",<br>"values": {<br>"type": "compound",<br>"valueLst": [<br>{ "statistic": "mean",<br>"type": "simple",<br>"unit": "mm^-2",<br>"values": [142.9, 178.5, 185.1, 215.8]<br>},<br>{ "statistic": "sd",<br>"type": "simple",<br>"unit": "mm^-2",<br>"values": [17.3, 26.4, 25.6, 10.1]<br>},<br>{ "statistic": "N",<br>"type": "simple",<br>"unit": "dimensionless",<br>"values": [7.0, 6.0, 7.0, 6.0]<br>}<br>]<br>}<br>},<br>"indepVars": [<br>{ "typeId": "BBP-002001",

```
            "values": {
              "statistic": "raw",
              "type": "simple",
              "unit": "day",
              "values": [10.0, 20.0, 30.0, 60.0]
            }
          }
        ],
        "type": "numericalTrace"
      },
      "id": "8873cc86-6f95-11e6-81bd-
64006a4c56ef",
      "isExperimentProperty": false,
      "requiredTags": [
        {  "id": "BBP_nlx_0022",
           "name": "Thalamic reticular nucleus cell -
GABAergic",
           "rootId": "NIFCELL:sao1813327414"
        },
        {  "id": "NIFGA:birnlex_1721",
           "name": "Thalamic reticular nucleus",
           "rootId": "NIFGA:birnlex_1167"
  }]}]
```


1. Connelly WM, Crunelli V, Errington AC. The Global Spike: Conserved Dendritic Properties Enable Unique Ca2+ Spike Generation in Low-Threshold Spiking Neurons. J Neurosci. 2015 Nov 25;35(47):15505–22.

2. Lee S-C, Patrick SL, Richardson KA, Connors BW. Two functionally distinct networks of gap junction-coupled inhibitory neurons in the thalamic reticular nucleus. J Neurosci Off J Soc Neurosci. 2014 Sep 24;34(39):13170–82.

3. Ohno S, Kuramoto E, Furuta T, Hioki H, Tanaka YR, Fujiyama F, et al. A Morphological Analysis of Thalamocortical Axon Fibers of Rat Posterior Thalamic Nuclei: A Single Neuron Tracing Study with Viral Vectors. Cereb Cortex. 2012 Dec 1;22(12):2840–57.

4. Martina M, Jonas P. Functional differences in Na+ channel gating between fast-spiking interneurones and principal neurones of rat hippocampus. J Physiol. 1997 Dec 15;505(Pt 3):593–603.

5. Cavdar S, Bay HH, Kirazli O, Cakmak YO, Onat F. Comparing GABAergic cell populations in the thalamic reticular nucleus of normal and genetic absence epilepsy rats from Strasbourg (GAERS). Neurol Sci Off J Ital Neurol Soc Ital Soc Clin Neurophysiol. 2013 Nov;34(11):1991–2000.

3 Supplementary Figures

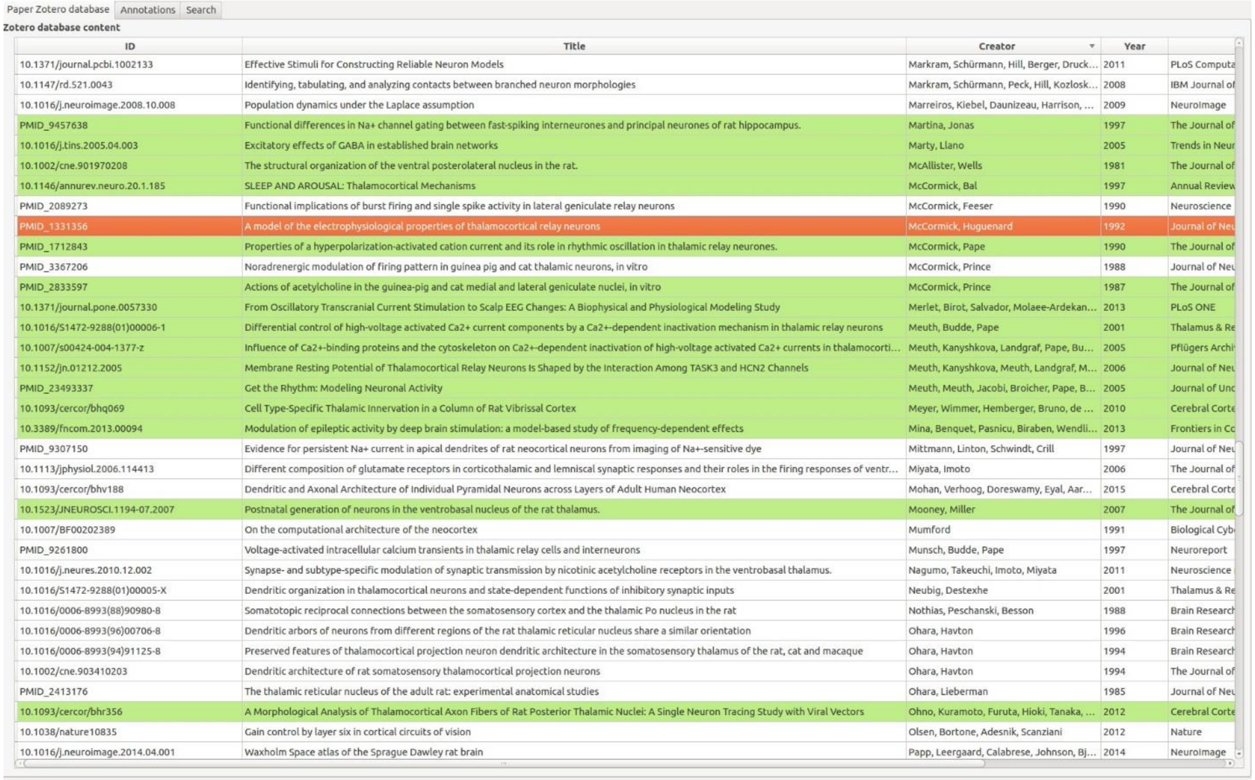

Paper Zotero database | Annotations | Search

Zotero database content

ID	Title	Creator	Year	
10.1371/journal.pcbi.1002133	Effective Stimuli for Constructing Reliable Neuron Models	Markram, Schürmann, Hill, Berger, Druck…	2011	PLoS Computa
10.1147/rd.521.0043	Identifying, tabulating, and analyzing contacts between branched neuron morphologies	Markram, Schürmann, Peck, Hill, Kozlosk…	2008	IBM Journal of
10.1016/j.neuroimage.2008.10.008	Population dynamics under the Laplace assumption	Marreiros, Kiebel, Daunizeau, Harrison, …	2009	NeuroImage
PMID_9457638	Functional differences in Na+ channel gating between fast-spiking interneurones and principal neurones of rat hippocampus.	Martina, Jonas	1997	The Journal of
10.1016/j.tins.2005.04.003	Excitatory effects of GABA in established brain networks	Marty, Llano	2005	Trends in Neur
10.1002/cne.901970208	The structural organization of the ventral posterolateral nucleus in the rat.	McAllister, Wells	1981	The Journal of
10.1146/annurev.neuro.20.1.185	SLEEP AND AROUSAL: Thalamocortical Mechanisms	McCormick, Bal	1997	Annual Review
PMID_2089273	Functional implications of burst firing and single spike activity in lateral geniculate relay neurons	McCormick, Feeser	1990	Neuroscience
PMID_1331356	A model of the electrophysiological properties of thalamocortical relay neurons	McCormick, Huguenard	1992	Journal of Neu
PMID_1712843	Properties of a hyperpolarization-activated cation current and its role in rhythmic oscillation in thalamic relay neurones.	McCormick, Pape	1990	The Journal of
PMID_3367206	Noradrenergic modulation of firing pattern in guinea pig and cat thalamic neurons, in vitro	McCormick, Prince	1988	Journal of Neu
PMID_2833597	Actions of acetylcholine in the guinea-pig and cat medial and lateral geniculate nuclei, in vitro	McCormick, Prince	1987	The Journal of
10.1371/journal.pone.0057330	From Oscillatory Transcranial Current Stimulation to Scalp EEG Changes: A Biophysical and Physiological Modeling Study	Merlet, Birot, Salvador, Molaee-Ardekan…	2013	PLoS ONE
10.1016/S1472-9288(01)00006-1	Differential control of high-voltage activated Ca2+ current components by a Ca2+-dependent inactivation mechanism in thalamic relay neurons	Meuth, Budde, Pape	2001	Thalamus & Re
10.1007/s00424-004-1377-z	Influence of Ca2+-binding proteins and the cytoskeleton on Ca2+-dependent inactivation of high-voltage activated Ca2+ currents in thalamocorti…	Meuth, Kanyshkova, Landgraf, Pape, Bu…	2005	Pflügers Archi
10.1152/jn.01212.2005	Membrane Resting Potential of Thalamocortical Relay Neurons Is Shaped by the Interaction Among TASK3 and HCN2 Channels	Meuth, Kanyshkova, Meuth, Landgraf, M…	2006	Journal of Neu
PMID_23493337	Get the Rhythm: Modeling Neuronal Activity	Meuth, Meuth, Jacobi, Broicher, Pape, B…	2005	Journal of Und
10.1093/cercor/bhq069	Cell Type-Specific Thalamic Innervation in a Column of Rat Vibrissal Cortex	Meyer, Wimmer, Hemberger, Bruno, de …	2010	Cerebral Corte
10.3389/fncom.2013.00094	Modulation of epileptic activity by deep brain stimulation: a model-based study of frequency-dependent effects	Mina, Benquet, Pasnicu, Biraben, Wendli…	2013	Frontiers in Co
PMID_9307150	Evidence for persistent Na+ current in apical dendrites of rat neocortical neurons from imaging of Na+-sensitive dye	Mittmann, Linton, Schwindt, Crill	1997	Journal of Neu
10.1113/jphysiol.2006.114413	Different composition of glutamate receptors in corticothalamic and lemniscal synaptic responses and their roles in the firing responses of ventr…	Miyata, Imoto	2006	The Journal of
10.1093/cercor/bhv188	Dendritic and Axonal Architecture of Individual Pyramidal Neurons across Layers of Adult Human Neocortex	Mohan, Verhoog, Doreswamy, Eyal, Aar…	2015	Cerebral Corte
10.1523/JNEUROSCI.1194-07.2007	Postnatal generation of neurons in the ventrobasal nucleus of the rat thalamus.	Mooney, Miller	2007	The Journal of
10.1007/BF00202389	On the computational architecture of the neocortex	Mumford	1991	Biological Cyb
PMID_9261800	Voltage-activated intracellular calcium transients in thalamic relay cells and interneurons	Munsch, Budde, Pape	1997	Neuroreport
10.1016/j.neures.2010.12.002	Synapse- and subtype-specific modulation of synaptic transmission by nicotinic acetylcholine receptors in the ventrobasal thalamus.	Nagumo, Takeuchi, Imoto, Miyata	2011	Neuroscience
10.1016/S1472-9288(01)00005-X	Dendritic organization in thalamocortical neurons and state-dependent functions of inhibitory synaptic inputs	Neubig, Destexhe	2001	Thalamus & Re
10.1016/0006-8993(88)90980-8	Somatotopic reciprocal connections between the somatosensory cortex and the thalamic Po nucleus in the rat	Nothias, Peschanski, Besson	1988	Brain Research
10.1016/0006-8993(96)00706-8	Dendritic arbors of neurons from different regions of the rat thalamic reticular nucleus share a similar orientation	Ohara, Havton	1996	Brain Research
10.1016/0006-8993(94)91125-8	Preserved features of thalamocortical projection neuron dendritic architecture in the somatosensory thalamus of the rat, cat and macaque	Ohara, Havton	1994	Brain Research
10.1002/cne.903410203	Dendritic architecture of rat somatosensory thalamocortical projection neurons	Ohara, Havton	1994	The Journal of
PMID_2413176	The thalamic reticular nucleus of the adult rat: experimental anatomical studies	Ohara, Lieberman	1985	Journal of Neu
10.1093/cercor/bhr356	A Morphological Analysis of Thalamocortical Axon Fibers of Rat Posterior Thalamic Nuclei: A Single Neuron Tracing Study with Viral Vectors	Ohno, Kuramoto, Furuta, Hioki, Tanaka, …	2012	Cerebral Corte
10.1038/nature10835	Gain control by layer six in cortical circuits of vision	Olsen, Bortone, Adesnik, Scanziani	2012	Nature
10.1016/j.neuroimage.2014.04.001	Waxholm Space atlas of the Sprague Dawley rat brain	Papp, Leergaard, Calabrese, Johnson, Bj…	2014	NeuroImage

Supplementary Figure S1. *Paper Zotero database* tab. It shows the publication references available in the Zotero library. Green records are those for which reference PDF and text files are available locally. Grey records (none are shown here) would indicate that the references are available on the server but not locally. White records are indicating publications for which no PDF files have been submitted to the server yet. The orange record indicates the selected publication. Corresponding annotations are loaded in the other panels of the interface.

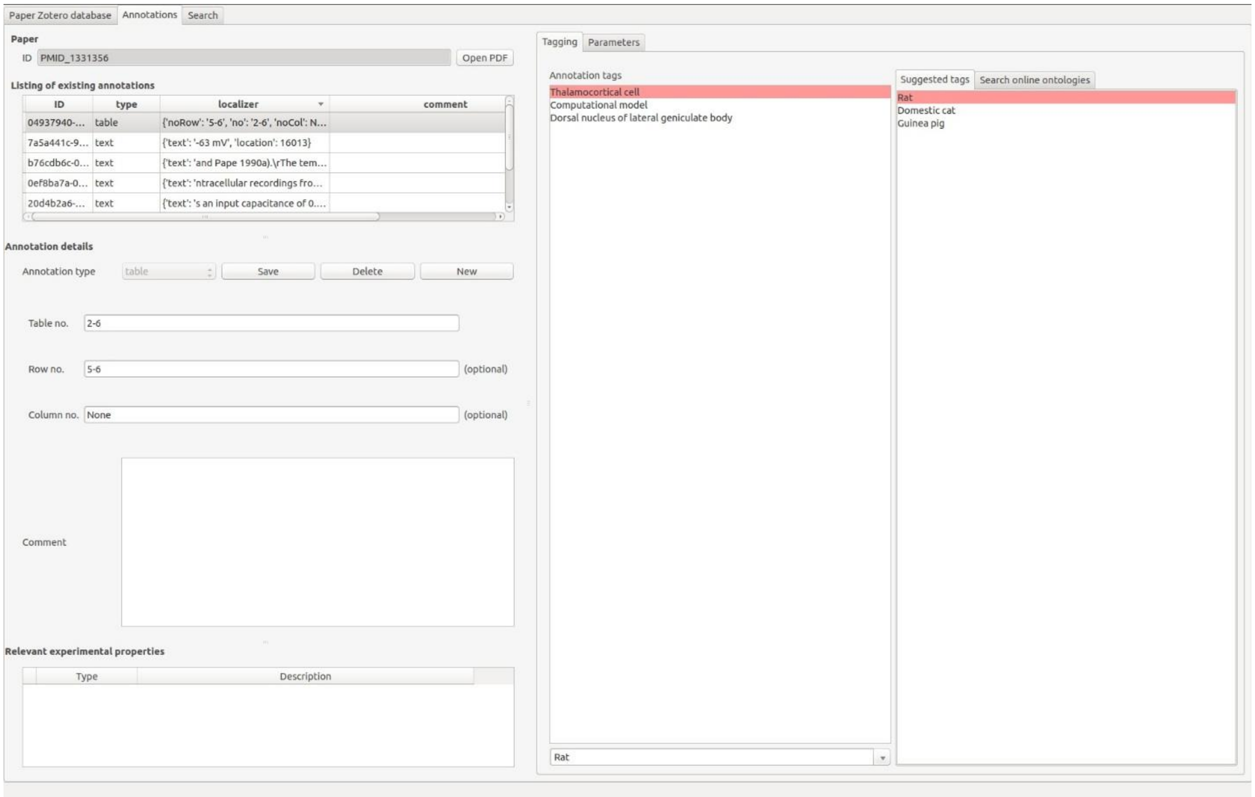

Supplementary Figure S2. *Annotations* tab. The upper left panel displays the list of annotations corresponding to the selected publication. The middle left panel contains the information related to the selected annotation. The lower left panel lists experimental properties annotated for this paper (none in this case). The right panel shows the tagging interface. The left widget lists the tags that have been selected for the loaded annotation. Left-clicking an item on this list while holding down the shift key turns the color of the tag from white to red (and vice versa). Red coloration in this list indicate that these tags that have been made “permanent”. This means that they will be added by default to any new annotation (for this publication only). Left-clicking on a tag on this list without holding the shift key simply removes the tag from the list. Tags can be added using the combobox situated below the list. This widget features an auto-completion list based on the terms already stored locally. Tags can also be selected by left-clicking on wanted terms in the right panel list. This widget lists suggested tags, ordered by frequency of past usage. Tags of this list can also be turned to red (as described for the first list), which will make them stick at the top of the list (for all publications). Finally, tags can also be selected by querying online ontologies. This can be performed in the *search online ontologies* tab (interface not shown here), which provides an auto-completed list of terms based on a query to the NIFSTD and NIP RESTful ontology services.

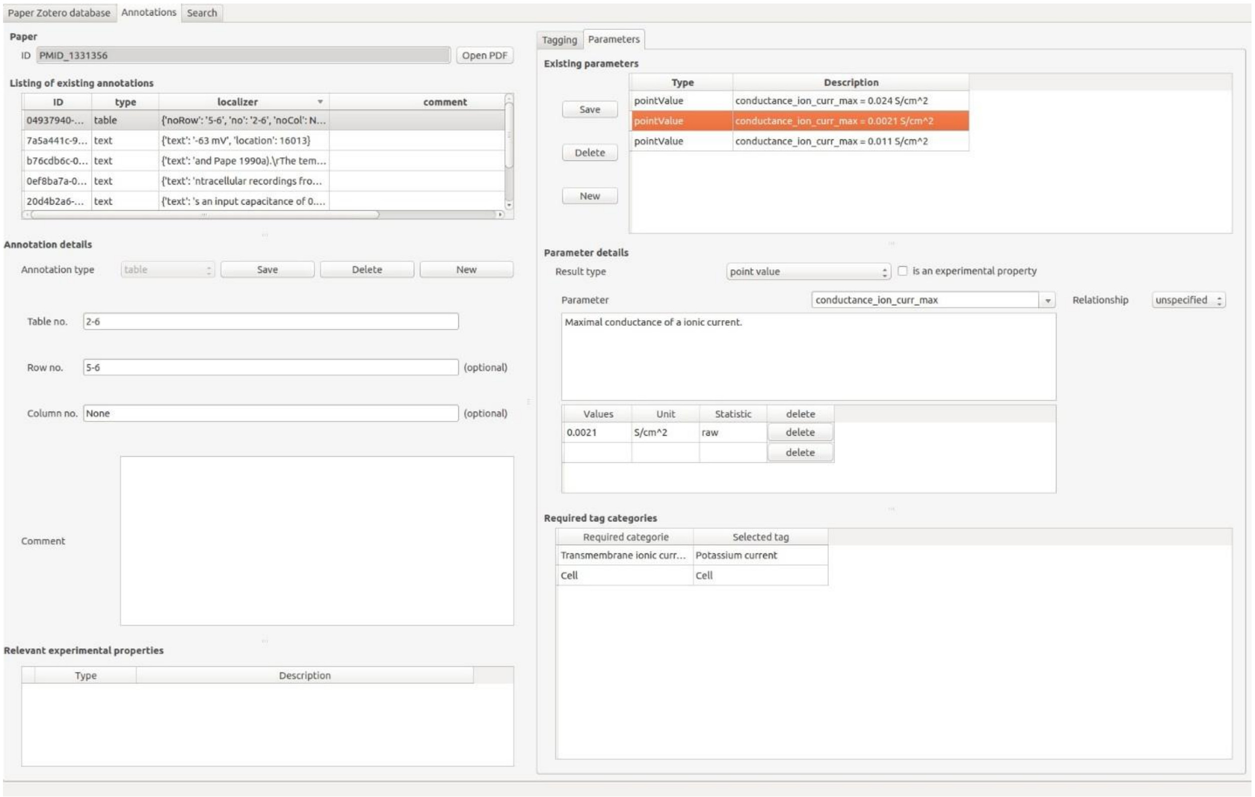

Supplementary Figure S3. Left panel: same as in Figure S2. Right panel: interface of the parameter annotation tab, which allows setting values to parameters contained in the annotation. Upper panel shows the list of created parameters, middle panel contains all information related to this parameter, and lower panel displays the chosen values for the required tags. The set of required tags is populated depending on the definition of the parameter type in the MPCV.

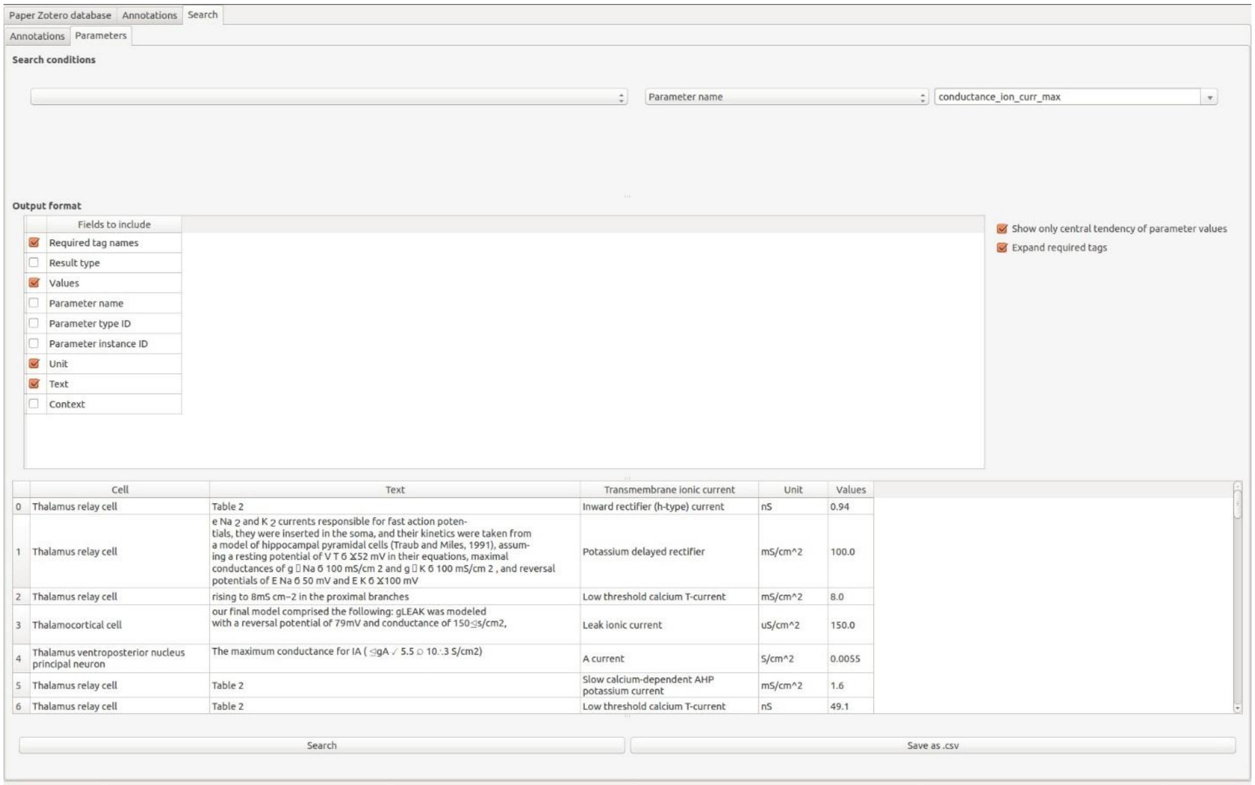

Supplementary Figure S4. *Search* tab. Both annotations and parameters can be searched. The upper panel allows setting the query which can be built as a hierarchy of AND, OR, and NOT clauses. For each clause, a searching index can be selected (e.g., parameter name, unit, author of the annotation) and a value can be set. The middle panel is used to format the output displayed in the lower panel. Double-clicking on a record of the output brings forward the annotation tab and loads the corresponding record. Results can be saved as CSV files.
